# Antimicrobial resistance profiles of *Escherichia coli* and prevalence of extended‐spectrum beta‐lactamase‐producing Enterobacteriaceae in calves from organic and conventional dairy farms in Switzerland

**DOI:** 10.1002/mbo3.1269

**Published:** 2022-03-16

**Authors:** Magdalena Nüesch‐Inderbinen, Claudia Hänni, Katrin Zurfluh, Sonja Hartnack, Roger Stephan

**Affiliations:** ^1^ Institute for Food Safety and Hygiene, Vetsuisse Faculty, University of Zurich Zurich Switzerland; ^2^ Section of Epidemiology, Vetsuisse Faculty, University of Zurich Zurich Switzerland

**Keywords:** antimicrobial resistance, calves, dairy farms, ESBL, *Escherichia coli*, organic

## Abstract

This study compared the antimicrobial resistance (AMR) among commensal *Escherichia coli* in the fecal microbiota of young calves raised on organic and on conventional dairy farms in Switzerland. Further, fecal carriage of extended‐spectrum beta‐lactamase (ESBL) producing Enterobacteriaceae was assessed for calves from both farming systems. Where possible, data on antimicrobial usage (AMU) were obtained. Antimicrobial susceptibility testing was performed on a total of 71 isolates using the disk diffusion method. ESBL producers were characterized by polymerase chain reaction‐based multilocus sequence typing and sequencing of the *bla*_ESBL_ genes. Organically raised calves were significantly more likely to harbor *E. coli* that showed AMR to ampicillin (odds ratio [OR]: 2.78, 95% confidence interval [CI]: 1.02–7.61, *p* = 0.046), streptomycin (OR: 3.22, 95% CI: 1.17–8.92, *p* = 0.046), kanamycin (OR: 11.3, 95% CI: 2.94–43.50, *p* < 0.001), and tetracycline (OR: 3.25, 95% CI: 1.13–9.31, *p* = 0.028). Calves with reported AMU were significantly more likely to harbor *E. coli* with resistance to ampicillin (OR: 3.91, 95% CI: 1.03–14.85, *p* = 0.045), streptomycin (OR: 4.35, 95% CI: 1.13–16.7, *p* = 0.045), and kanamycin (OR: 8.69, 95% CI: 2.01–37.7, *p* = 0.004). ESBL‐producing Enterobacteriaceae (18 *E. coli* and 3 Citrobacter braakii) were detected exclusively among samples from conventionally farmed calves (OR: infinity [∞], 95% CI: 2.3–∞, *p* < 0.0013). The observations from this study suggest that AMR is highly prevalent among commensal *E. coli* in young dairy calves, irrespective of the farm management system, with proportions of certain resistance phenotypes higher among organic calves. By contrast, the occurrence of ESBL producers among young dairy calves may be linked to factors associated with conventional farming.

## Graphical abstract

This study compared antimicrobial resistance (AMR) among commensal *Escherichia coli* of young calves raised on organic and on conventional dairy farms in Switzerland. Proportions of AMR were higher among organically raised calves and calves with antibiotic usage. Extended‐spectrum β‐lactamase (ESBL)‐producing Enterobacteriaceae were found exclusively among calves from conventional dairy farms.

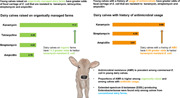

## 1 INTRODUCTION

Antimicrobial resistance (AMR) is a major public health care issue that is increasingly reflected in veterinary medicine. Also in veterinary medicine, adequate antimicrobial treatment of bacterial infections is necessary to promote the health and welfare of animals. However, it is widely acknowledged that the use, overuse, and misuse of antimicrobial agents promote the emergence of resistant bacteria and the dissemination of AMR genes. In food‐producing animals, the use of antimicrobials comes with the risk of spreading AMR at the animal/human interface, through the food chain, or by contamination of the farm environment (Carattoli, 2008; Nüesch‐Inderbinen & Stephan, 2016). Switzerland, like other European countries (World Health Organization, 2011), has developed and implemented a national strategy on antibiotic resistance (StAR) to address the threat of AMR affecting the human and animal health sectors and the environment (Swiss Federal Council, 2015). Data on the prevalence of AMR in zoonotic and indicator bacteria isolated from humans, livestock, and food are published regularly in the Swiss Antibiotic Resistance Report (Federal Office of Public Health and Federal Food Safety and Veterinary Office, 2020). In 2019, indicator *Escherichia coli* isolated by nonselective methods from caecal content samples of healthy slaughter calves in Switzerland were most commonly resistant to tetracyclines (36.2%), sulfonamides (31.2%), ampicillin (26.1%), trimethoprim (13.1%), and chloramphenicol (7.0%). Applying selective enrichment methods, 32.9% of sampled calves revealed *E. coli* with resistance to 3rd and/or 4th generation cephalosporins, indicating the presence of extended‐spectrum beta‐lactamase‐(ESBL) or plasmid‐mediated beta‐lactamase (pAmpC) producing bacteria (Federal Office of Public Health and Federal Food Safety and Veterinary Office, 2020). ESBL‐producing Enterobacteriaceae are of particular concern since 3rd and 4th generation cephalosporins are critically important antimicrobials for use in humans (World Health Organization, 2019). Data provided by the Federal Food Safety and Veterinary Office (FSVO) indicate that the usage of antimicrobials in livestock decreased by 32% between 2015 and 2020 (Federal Food Safety and Veterinary Office, 2019). However, despite efforts to minimize the usage of antimicrobials in Swiss livestock, the prevalence of ESBL producers in slaughter calves has remained on a high level (>30%) since 2015, indicating that additional factors such as differences in farm management systems may play a role in their occurrence (Federal Office of Public Health and Federal Food Safety and Veterinary Office, 2020). In the EU regulation for organic dairy herds, antimicrobial therapy is restricted to three treatments per individual cow and year (Council Regulation [EC] No 834/2007 and EC No 889/2008). In Switzerland, the Swiss Ordinance on Organic Farming (SR 910.18) allows antimicrobial agents to be prescribed by a veterinarian only if homeopathic or phytotherapeutic products failed to prevent suffering or distress to the animal. Furthermore, the ordinance requires twice the legal withdrawal period for organically produced foodstuffs from treated animals. Animals that receive more than three courses of antimicrobial treatments within 1 year may no longer be classified as organically farmed. However, it is unclear whether this set of regulations results in a lower prevalence of AMR in organic dairy calves and there are currently no data that compare AMR among dairy calves reared in organic and conventional productions systems in Switzerland. Therefore, it was the aim of this study to assess the prevalence of AMR *E. coli* and the occurrence of ESBL‐producing Enterobacteriaceae in young dairy calves on their birth farms and to evaluate any differences between calves from organically and conventionally managed dairy farms.

## 2 MATERIALS AND METHODS

### 2.1 Fecal sampling

During September 2020, a total of 24 officially registered organic, and 30 conventional dairy farms were visited throughout four cantons in the northwest region of Switzerland. Visits were conducted with the approval of the farmers. Only calves that were born on the respective farm were included in the study. Where available, data on antimicrobial usage (AMU) was recorded. In the current study, AMU included antimicrobial treatment of the calves or feeding of discard milk from cows treated with antibiotics.

The age of the calves ranged from 2 to 120 days. To ensure a noninvasive procedure, fresh feces were collected from pen floors and animal enclosures using plastic bags. A total of 196 samples were collected and placed in cooler boxes for transport and stored at −20°C until processing.

Before microbiological analysis, the samples were thawed at 4°C overnight. A sterile cotton swab of each sample was placed in a sterile blender bag (Seward), homogenized at a 1:10 ratio in Enterobacteriaceae enrichment (EE) broth (BD), and incubated at 37°C for 24 h.

### 2.2 Isolation of *E. coli*

For isolation of *E. coli*, one loopful of each of the EE cultures was streaked onto Rapid'*E. coli* two agar plates (Bio‐Rad Laboratories) and incubated at 37°C for 24 h. From each plate, one single *E. coli* colony was subcultured on nonselective Plate Count (PC) agar (Bio‐Rad) and incubated at 37°C for 24 h.

### 2.3 Screening for ESBL‐producers

For the detection of ESBL‐producing Enterobacteriaceae, one loopful of each of the EE cultures was streaked onto Brilliance ESBL^TM^ agar plates (Oxoid). Plates were incubated under aerobic conditions at 37°C for 24 h. Colonies with different coloration were subcultured on Brilliance^TM^ ESBL agar plates at 37°C for 24 h. From each plate, single colonies were picked and subcultured on PC agar for 24 h at 37°C.

Species were identified using matrix‐assisted laser desorption ionization‐time of flight mass spectrometry (MALDI‐TOF‐MS; Bruker Daltonics).

### 2.4 Antimicrobial susceptibility testing (AST)

AST was performed using the disk diffusion method according to the guidelines of the Clinical and Laboratory Standards Institute (Clinical and Laboratory Standards Institute, 2020). Antimicrobial substances included ampicillin, amoxicillin/clavulanic acid, cefazolin, cefotaxime, cefepime, nalidixic acid, ciprofloxacin, sulfamethoxazole‐trimethoprim, fosfomycin, azithromycin, nitrofurantoin, streptomycin, kanamycin, gentamicin, chloramphenicol, and tetracycline (Becton, Dickinson). Results were interpreted according to CLSI breakpoints for human clinical isolates (Clinical and Laboratory Standards Institute, 2020). In the absence of clinical breakpoints for azithromycin resistance in *Citrobacter* spp. and *E. coli*, a zone diameter of ≤12 mm was interpreted as resistant, based on data reported by Meerwein et al. (2020).

### 2.5 Identification of *bla*_ESBL_ genes

DNA of ESBL‐producers was extracted using a standard heat lysis protocol. Screening for *bla*_TEM_ and *bla*_SHV_ was carried out using primers described previously (Pitout et al., 1998). Screening for *bla*_CTX‐M_ alleles belonging to CTX‐M groups 1, 2, 8, 9, and 25 was performed as described by Woodford et al. (2006). Amplicons for sequencing *bla*_CTX‐M_ genes were generated using primers described previously (Geser et al., 2012). Synthesis of primers and DNA custom sequencing was carried out by Microsynth (Balgach). Nucleotide sequences were analyzed with CLC Main Workbench 20.0.4 (Qiagen). For database searches, the BLASTN program of NCBI (http://www.ncbi.nlm.nih.gov/blast/) was used.

### 2.6 Phylogenetic analysis and multilocus sequence typing (MLST) of ESBL producing *E. coli*

The distribution of phylogenetic groups among the ESBL‐producing *E. coli* was determined by polymerase chain reaction (PCR) targeting the genes *chuA, yjaA, arpA*, and TspE4.C2, as described by Clermont et al. (2013). Isolates were thereby assigned to one of the eight phylogenetic groups A, B1, B2, C, D, E, F (*E. coli* sensu stricto), or *Escherichia* clade I.

For MLST of *E. coli* isolates, internal fragments of the seven housekeeping genes (*adk, fumC, gyrB, icd, mdh, purA*, and *recA*) were amplified by PCR as described by Wirth et al. (2006). The amplification products were custom sequenced and sequence types (STs) were determined using the *E. coli* MLST database website (https://enterobase.warwick.ac.uk).

### 2.7 Statistical analysis

For the descriptive statistical analysis, 95% binomial confidence intervals (CI) were obtained for the proportions of antibiotic resistance and ESBL‐producers with the command BinomCI specifying the type “Jeffreys” in the package DescTools (Signorell  et al., 2020), using R (R Core Team, 2021). To assess if antibiotic resistance is significantly associated with farming type or AMU, generalized linear mixed models were applied for all antibiotics where at least 10% of the isolates were resistant. The analysis was performed with the packages “geepack” (Halekoh et al., 2006) “lme4” (Bates et al., 2015), and “MASS” (Venables & Ripley, 2002). Model selection was based on likelihood ratio tests with the package “lmtest” (Hothorn et al., 2018). To account for potential within‐herd clustering, farms were included as random effects.

The prevalence of ESBL‐producers at the farm level was compared with the two‐tailed Fisher's exact test.

## 3 RESULTS

### 3.1 Sampling

This study included 196 calves from 24 organic and 30 conventional dairy farms. The number of sampled calves per farm varied between one and five, with a median of four calves per farm. Overall, 87 fecal samples were collected from organic, and 109 from conventional dairy farms.

AMU was noted for 17 calves. Seven calves were from organic farms and 10 from conventional farms. Among the seven calves from organically managed farms, five had been fed discard milk from cows treated with antibiotics for mastitis or other illnesses, one had a history of treatment with tetracycline, and one had received an aminoglycoside. Of the 10 calves from conventional farms, two had been fed discard milk, four had a history of treatment with tetracycline, three had received a penicillin‐streptomycin combination, and one calf had florfenicol.

### 3.2 Antimicrobial susceptibility of *E. coli* isolates

Using Rapid'*E. coli* agar, *E. coli* was recovered from all 196 fecal samples. For further analysis, a total of 71 isolates were selected for AST. Thereof, 54 originated from calves without AMU (24 *E. coli* from organically farmed calves and 30 *E. coli* from conventionally farmed calves), representing one isolate per farm. In addition, 17 *E. coli* from calves with a history of AMU were included (seven isolates from four organic farms, and 10 isolates from six conventional dairies).

Overall, the most common resistances were to ampicillin (34/71, 48%), streptomycin (33/71, 46%), tetracycline (31/71, 45%), kanamycin (23/71, 32%), and chlorampheicol (19/71, 27%).

### 3.3 Descriptive analysis

**Figure 1:**
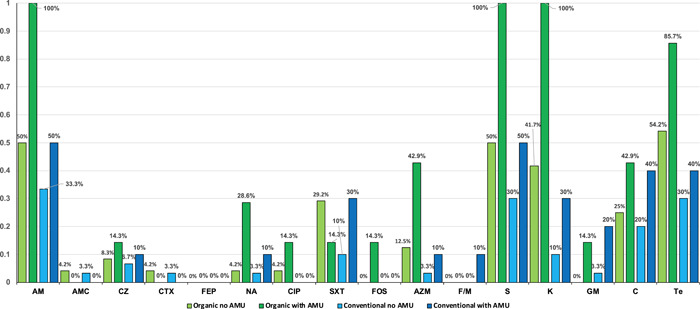
Proportions of antimicrobial resistance (AMR) to 16 antimicrobial agents among 71 *Escherichia coli* from fecal samples of calves from 24 organic and 30 conventional Swiss dairy farms. *E. coli* were isolated from samples from organically raised calves (31 isolates) and conventionally raised calves (40 isolates). Columns in light green indicate the proportion of resistant *E. coli* from organic calves without recorded antimicrobial usage (AMU), dark green columns represent *E. coli* from organic calves with AMU. Columns in light blue show values for *E. coli* from conventionally raised calves without AMU, dark blue columns indicate *E. coli* from conventional calves with AMU. Data labels show percentage values. AM, ampicillin; AMC, amoxicillin/clavulanic acid; AZM, azithromycin; C, chloramphenicol; CIP, ciprofloxacin; CTX, cefotaxime; CZ, cefazolin; FEP, cefepime; F/M, nitrofurantoin; FOS, fosfomycin; GM, gentamicin; K, kanamycin; NA, nalidixic acid; S, streptomycin; SXT, sulfamethoxazole‐trimethoprim; Te, tetracycline

Figure 1 presents the proportions of resistant *E. coli* according to farming type and AMU. Except for cefepime, fosfomycin, nitrofurantoin, and gentamicin, all antibiotics tested showed higher proportions of resistant *E. coli* from organic calves without AMU, which were most prominent for ampicillin, sulfamethoxazole/trimethoprim, azithromycin, kanamycin, streptomycin, and tetracycline. Apart from amoxicillin/clavulanic acid, cefotaxime, cefepime nitrofurantoin, and gentamicin, the proportion of resistance to *E. coli* was also higher among samples from organic calves with a history of AMU than among *E. coli* from conventionally raised calves with AMU. Table 1 presents the proportions of resistant *E. coli* according to farming type and AMU.

**Table 1:** Results of descriptive statistical analysis and generalized mixed models of the distribution of antimicrobial‐resistant *Escherichia coli* from feces of calves from organic and from conventional dairy farms and calves with and without antimicrobial usage

	Descriptive analysis					Mixed model	
		Farming type		Antimicrobial usage (AMU)		Farming type	AMU
Antimicrobial substance		Organic (*n* = 31)	Conventional (*n* = 40)	Yes (*n* = 17)	No (*n* = 54)		
Ampicillin	*n* (proportion)	19 (0.61)	15 (0.38)	12 (0.71)	22 (0.41)	–	–
	[95% CI]	[0.44–0.77]	[0.24–0.53]	[0.47–0.88]	[0.28–0.54]	[1.02–7.61]	[1.03–14.85]
	*p* value (OR)	–	–	–	–	**0.046** (2.78)	**0.045** (3.91)
Amoxicillin/clavulanic acid	*n* (proportion)	3 (0.1)	3 (0.08)	2 (0.12)	4 (0.07)		
	[95% CI]	[0.03–0.24]	[0.02–0.19]	[0.025–0.33]	[0.03–0.17]	[0.14–6.16]	[0.11–1.74]
	*p* value (OR)	–	–	–	–	0.942 (0.93)	0.25 (0.45)
Cefazolin	*n* (proportion)	1 (0.03)	1 (0.03)	0 (0)	2 (0.04)	–	–
	[95% CI]	[0.003–0.14]	0.003–0.11]	[0–0.135]	[0.008–0.12]	–	–
	*p* value (OR)	–	–	–	–	–	
Cefotaxime	*n* (proportion)	1 (0.03)	1 (0.03)	0 (0)	2 (0.04)	–	–
	[95% CI]	[0.004–0.14]	[0.003–0.11]	[0–0.14]	[0.008–0.12]	–	–
	*p* value (OR)	–	–	–	–	–	–
Cefepime	*n* (proportion)	0 (0)	0 (0)	0 (0)	0 (0)	–	–
	[95% CI]	[0–0.077]	[0–0.06]	[0–0.135]	[0–0.045]	–	–
	*p* value (OR)	–	–	–	–	–	–
Nalidixic acid	*n* (proportion)	3 (0.1)	2 (0.05)	3 (0.18)	2 (0.04)	–	–
	[95% CI]	[0.3–0.23]	[0.01–0.15]	[0.05–0.40]	[0.008–0.12]	–	–
	*p* value (OR)	–	–	–	–		
Ciprofloxacin	*n* (proportion)	2 (0.06)	0 (0)	1 (0.06)	1 (0.02)	–	–
	[95% CI]	[0.013–0.2]	[0–0.06]	[0.006–0.24]	[0.002–0.08]	–	–
	*p* value (OR)	–	–	–	–	–	
Sulfamethoxazole/trimethoprim	*n* (proportion)	8 (0.26)	6 (0.15)	4 (0.24)	10 (0.19)	–	–
	[95% CI]	[0.13–0.43]	[0.06–0.28]	[0.09–0.47]	[0.11–0.32]	[0.744–11.2]	[0.785–2.93]
	*p* value (OR)	–	–	–	–	0.125 (2.89)	0.214 (1.52)
Fosfomycin	*n* (proportion)	1 (0.03)	0 (0)	1 (0.06)	0 (0)	–	–
	[95% CI]	[0.003–0.14]	[0–0.06]	[0.006–0.24]	[0–0.05]	–	–
	*p* value (OR)	–	–	–	–	–	–
Azithromycin	*n* (proportion)	6 (0.19)	2 (0.05)	4 (0.24)	4 (0.07)	–	–
	[95% CI]	[0.85–0.35]	[0.01–0.15]	[0.09–0.47]	[0.026–0.17]	[0.8–35.1]	[0.499–35.5]
	*p* value (OR)	–	–	–	–	0.084 (5.3)	0.186 (4.21)
Nitrofurantoin	*n* (proportion)	0 (0)	1 (0.03)	1 (0.06)	0 (0)	–	–
	[95% CI]	[0–0.07]	[0.003–0.11]	[0.006–0.24]	[0–0.045]	–	–
	*p* value (OR)	–	–	–	–	–	–
Streptomycin	*n* (proportion)	19 (0.61)	14 (0.35)	12 (0.71)	21 (0.39)	–	–
	[95% CI]	[0.43–0.77]	[0.21–0.5]	[0.47–0.88]	[0.33–0.61]	[1.17–8.92]	[1.13–16.7]
	*p* value (OR)	–	–	–	–	**0.046** (3.22)	**0.045** (4.35)
Kanamycin	*n* (proportion)	17 (0.55)	6 (0.15)	10 (0.59)	13 (0.24)	–	–
	[95% CI]	[0.37–0.71]	[0.06–0.28]	[0.36–0.79]	[0.15–0.39]	[2.94–43.5]	[2.01–37.7]
	*p* value (OR)	–	–	–	–	**<0.001** (11.3)	**0.004** (8.69)
Gentamicin	*n* (proportion)	2 (0.06)	3 (0.08)	3 (0.18)	2 (0.04)	–	–
	[95% CI]	[0.01–0.19]	[0.02–0.18]	[0.05–0.4]	[0.008–0.12]	–	–
	*p* value (OR)	–	–	–	–	–	–
Chloramphenicol	*n* (proportion)	9 (0.29)	10 (0.25)	7 (0.41)	12 (0.22)	–	–
	[95% CI]	[0.15–0.46]	[0.13–0.39]	[0.2–0.64]	[0.14–0.4]	[0.383–4.06]	[0.639–5.77]
	*p* value (OR)	–	–	–	–	0.714 (1.25)	0.246 (1.92)
Tetracycline	*n* (proportion)	19 (0.61)	13 (0.33)	10 (0.59)	22 (0.41)	–	–
	[95% CI]	[0.44–0.76]	[0.19–0.48]	[0.36–0.8]	[0.35–0.63]	[1.13–9.31]	[0.656–8.28]
	*p* value (OR)	–	–	–	–	**0.028** (3.25)	0.191 (2.33)

*Note*: *p* ≤ 0.05 are indicated in bold; –, not applicable.

Abbreviations: CI, confidence interval; OR, odds ratio.

### 3.4 Mixed models

The mixed model estimate indicated significant differences in resistance to ampicillin (odds ratio [OR]: 2.78, 95% confidence interval [CI]: 1.02–7.61, *p* = 0.046,), streptomycin (OR: 3.22, 95% CI: 1.17–8.92, *p* = 0.046) kanamycin (OR: 11.3, 95% CI: 2.94–43.5, *p* < 0.001), and tetracycline (OR: 3.25, 95% CI: 1.13–9.31, *p* = 0.028) between *E. coli* from organically farmed and conventionally farmed calves (Table 1). For *E. coli* from calves with reported AMU, the proportions of *E. coli* showing resistance to ampicillin (OR: 3.91, 95% CI: 1.03–14.85, *p* = 0.045), streptomycin (OR: 4.35, 95% CI: 1.13–16.7, *p* = 0.045), and kanamycin (OR: 8.69, 95% CI: 2.01–37.7, *p* = 0.004) varied significantly between the two groups (Table 1).

### 3.5 ESBL‐producing Enterobacteriaceae

ESBL‐producers were isolated from 21 (11%) of the 196 fecal samples and were identified on 10 (33%) of the 30 conventional dairy farms and none of the organic farms (OR infinity [∞], 95% CI: 2.3–∞, *p* < 0.0013). Of the 10 conventional farms, six (20%) had two or more calves shedding ESBL‐producers (Table 2). Overall, 18 *E. coli* and three *Citrobacter braakii* were recovered.

**Table 2:** Characteristics of extended‐spectrum β‐lactamase (ESBL)‐producing *Escherichia coli* and *Citrobacter braakii* from feces of calves with or without antimicrobial usage from Swiss conventional dairy farms

Sample ID	Farm ID	Species	PG	ST (CC)	ESBL	Resistance profile	AMU
BB1E	BB	*Escherichia coli*	A	540 (–)	CTX‐M‐1	AM, AMP, CTX, SXT, S, K, GM, C, Te	No
BB2E	BB	*E. coli*	A	540 (–)	CTX‐M‐1	AM, AMP, CTX, SXT, S, K, GM, C, Te	No
CC2E	CC	*E. coli*	A	540 (–)	CTX‐M‐1	AM, AMP, CTX, SXT, S, K, GM, C, Te	No
DD4E	DD	*E. coli*	B1	58 (155)	CTX‐M‐1	AM, AMP, CTX, SXT,	No
AA1E	AA	*E. coli*	B1	58 (155)	CTX‐M‐3	AM, AMP, CTX,	No
AA2E	AA	*E. coli*	B1	711 (–)	CTX‐M‐3	AM, AMP, CTX,	No
AA3E	AA	*E. coli*	A	1434 (10)	CTX‐M‐3	AM, AMP, CTX, K, C, Te	No
AA4E	AA	*E. coli*	C	88 (23)	CTX‐M‐3	AM, AMP, CTX, FEP, SXT, S, K, Te	No
E2E	E	*E. coli*	A	761 (10)	CTX‐M‐14	AM, AMP, CTX, SXT, S, K, C, Te	No
E3E	E	*E. coli*	A	761 (10)	CTX‐M‐14	AM, AMP, CTX, SXT, S, K, C, Te	No
C1E	C	*E. coli*	A	10 (10)	CTX‐M‐15	AM, AMP, CTX, NAL, CIP, SXT, AZM, S, K, C, Te	Yes^a^
C2E	C	*E. coli*	A	10 (10)	CTX‐M‐15	AM, AMP, CTX, NAL, CIP, SXT, AZM, S, K, C, Te	Yes^a^
C3E	C	*E. coli*	A	10 (10)	CTX‐M‐15	AM, AMP, CTX, NAL, CIP, SXT, AZM, S, K, C, Te	Yes^a^
F1E	F	*E. coli*	A	10 (10)	CTX‐M‐15	AM, AMP, CTX, S, K, GM, Te	No
G1E	G	*E. coli*	A	10 (10)	CTX‐M‐15	AM, AMP, CTX, FEP, S, K, GM, Te	No
G2E	G	*E. coli*	A	10 (10)	CTX‐M‐15	AM, AMP, CTX, NAL, CIP, SXT, AZM, S, K, GM, C, Te	No
G4E	G	*E. coli*	A	10 (10)	CTX‐M‐15	AM, AMP, CTX, NAL, CIP, SXT, AZM, S, K, C, Te	No
W1E	W	*E. coli*	B1	906 (–)	CTX‐M‐15	AM, AMP, CTX, FEP, SXT, S, K, GM, C, Te	No
U1E	U	*Citrobacter braakii*	–	–	CTX‐M‐1	AM, AMP, CTX, SXT, S, K, GM,	No
U3.1E	U	*C. braakii*	–	–	CTX‐M‐1	AM, AMP, CTX, FEP, SXT, S, K, GM,	No
U5E	U	*C. braakii*	–	–	CTX‐M‐1	AM, AMP, CTX, SXT, S, K, GM,	No

Abbreviations: AM, ampicillin; AMP, amoxicillin/clavulanic acid; AMU, antimicrobial usage; AZM, azithromycin; C, chloramphenicol; CC, clonal complex; CIP, ciprofloxacin; CTX, cefotaxime; CZ, cefazolin; FEP, cefepime; GM, gentamicin; K, kanamycin; NAL, nalidixic acid; PG, phylogenetic group; S, streptomycin; ST, sequence type; SXT, sulfamethoxazole‐trimethoprim; Te, tetracycline; –, not applicable.

^a^ Treatment with penicillin‐streptomycin was recorded.

Of the 18 ESBL‐producing *E. coli*, four isolates from three different farms harbored *bla*_CTX‐M‐1_, four isolates originating from the same farm harbored *bla*_CTX‐M‐3_, two *E. coli* from one farm contained *bla*_CTX‐M‐14_, and eight isolates from four farms carried *bla*_CTX‐M‐15_ (Table 2). Fifteen (83%) of the ESBL‐producing *E. coli* were MDR (Table 2).

Phylogenetic classification allocated 13 *E. coli* to phylogenetic Group A and four to phylogenetic Group B1, both of which typically contain commensal strains. One strain belonged to phylogenetic Group C (Table 2).

MLST identified eight different *E. coli* STs: ST10 (*n* = 7), ST540 (*n* = 3), ST58 (*n* = 2), ST761 (*n* = 2), ST88 (*n* = 1), ST711 (*n* = 1), ST906 (*n* = 1), and ST1434 (*n* = 1). Overall, 10 *E. coli* belonged to clonal complex (C) 10 (Table 2). Two *E. coli* ST58 were assigned to CC155.

AMU was known in three cases of calves harboring CTX‐M‐15‐producing *E. coli*, all of which had received penicillin‐streptomycin and were from the same farm (Table 2). The three *C. braakii* harbored *bla*_CTX‐M‐1_ and were isolated from calves from one farm (Table 2).

## 4 DISCUSSION

*E. coli* is an important indicator organism for monitoring AMR in food‐producing animals and AMR in *E. coli* is thought to reflect the AMU at the farm level in different animal production sectors (Caruso, 2018; Schönecker et al., 2019). Decreasing temporal trends in AMR among commensal *E. coli* from fattening calves at slaughter in Switzerland between 2017 and 2019 coincide with policies aimed at reducing the use of veterinary antimicrobials (Federal Food Safety and Veterinary Office, 2019). There is a growing level of public awareness of the importance of antimicrobial stewardship in animal farming, and many consumers associate reduced antibiotic use and improved animal welfare with organic farming (Clark et al., 2016; Rell et al., 2020). There is however no data that assess AMR among *E. coli* from dairy calves at the farm level or studies that compare AMR prevalence in different production systems in Switzerland.

In this study, for some antimicrobials such as ampicillin, tetracycline, and chloramphenicol, resistance percentages for commensal *E. coli* from dairy calves were higher (48%, 45%, and 27%, respectively) than data reported for fattening calves at slaughter in Switzerland in 2019 (26.1%, 36.2%, and 7%, respectively; Federal Food Safety and Veterinary Office, 2019). These findings are in agreement with previous reports on elevated AMR prevalence among *E. coli* in young calves which decreases with the age of the animals (Haley et al., 2020; Hoyle et al., 2004; Khachatryan et al., 2004). Sampling older animals may therefore underestimate AMR at the herd level on dairy farms.

Overall, the data presented in this study indicate that calves from organically managed farms are more likely than those from conventional farms to harbor *E. coli* showing resistance to ampicillin, streptomycin, kanamycin, and tetracycline. These results were unexpected, considering that organic farmers are obliged to apply a more restrictive AMU policy compared to conventional farmers. The results are dissimilar to previous studies from European countries that report little or no difference and in contrast to data from the USA that indicate a significantly lower prevalence, of AMR in *E. coli* isolates from calves on organic dairy farms compared to conventionally produced animals (Sato et al., 2005; Sjöström et al., 2020; Wilhelm et al., 2009). In view of organic farming in other livestock sectors, it is worth mentioning that on organic broiler farms, AMR commensal *E. coli* are still common, albeit less frequent than on conventional farms (Musa et al., 2020; Pesciaroli et al., 2020). However, organic production regulations, AMU, as well as study designs and methodologies vary between studies and should be considered when comparing data (Wilhelm et al., 2009).

The possible reasons for the unexpected prevalence of AMR among organically managed calves in the present study may include differences in feed intake or exposure to AMR *E. coli* through the environment. Environmental differences between organic and conventional farms may involve the possibility of free‐ranging or access to pasturage. Notably, the proportion of treated animals in both groups was similar, which may explain the unanticipated prevalence of AMR in organic calves. However, further investigations that include higher sample numbers and additional risk factors are needed to confirm and explain our observations.

Our data further indicate that in young calves on dairy farms, AMR *E. coli* are present in calves irrespective of apparent AMU. Similar observations have been reported in earlier studies that suggest that in addition to exposure to antimicrobials, environmental and host factors may be associated with the selection of AMR in young calves (Haley et al., 2020). Drivers that select for AMR in young dairy calves are currently poorly understood and warrant further investigation (Haley & Van Kessel, 2021). Our data further suggest that calves with AMU from both farming types are more likely to carry ampicillin‐, streptomycin‐, and kanamycin‐resistant *E. coli* than calves without AMU.

In contrast to AMR commensal *E. coli*, ESBL‐producing Enterobacteriaceae were detected exclusively among calves reared on conventionally managed dairy farms.

With a prevalence of 33% at the farm level, our data correspond to the herd prevalence of 32% reported for Swiss fattening calves at slaughter (Federal Food Safety and Veterinary Office, 2019). The similar prevalences among the two age groups suggest that ESBL producers may be maintained at a high level in the microbiota of calves during the fattening period. The absence of ESBL producers among organically reared calves confirms recent data from a comparable study from Sweden that examined the prevalence of ESBL producing *E. coli* in calves from organic and conventional dairy farms (Sjöström et al., 2020), but the contrast with a previous study from the Netherlands which reported that 11% of organic dairy herds were positive for ESBL producers (Santman‐Berends et al., 2017). However, while the study by Sjöström et al. (2020) as well as the present study used healthy young calves as an indicator for AMR and ESBL producers in organic and conventional dairy herds, Santman‐Berends et al. (2017) determined ESBL herd status based on the bacteriological culture result of slurry sample, which should be taken into account when comparing results.

ESBL dairy herd status is significantly associated with the treatment of cases of clinical mastitis, a high proportion of treated calves, and the use of 3rd and/or 4th generation cephalosporins (Gonggrijp et al., 2016; Lam et al., 2020). However, for the present study, no further information was available on the prescribed antimicrobials and treatment of the other animals on the farms, and we found no indication of 3rd and/or 4th generation cephalosporin usage that would explain the ESBL herd status of the conventional dairy farms analyzed in this study. Therefore, while there is currently still a lack of data in the scientific literature to confirm our findings, the results from our study indicate that organic farming systems may contribute to preventing or reducing the prevalence of ESBL‐producing *E. coli* among young calves on dairy farms.

Notably, our findings differ from results obtained from studies on poultry farms, where ESBL‐producers show a high prevalence both in conventional and organic poultry farming (Saliu et al., 2017). In both poultry farming systems, vertical transmission of plasmids within the production pyramid rather than the clonal spread of certain lineages account for the occurrence and long‐term maintenance of ESBL‐producers among broilers (van Hoek et al., 2018; Zurfluh et al., 2014).

In this study, the most frequently observed ESBL variants were CTX‐M‐15 which is the most important ESBL in human medicine (Cantón et al., 2012), and CTX‐M‐1 which represents the predominant ESBL subtype in Enterobacteriaceae from livestock in Europe (Ewers et al., 2012). *E. coli* ST10 harboring *bla*_CTX‐M‐15_ was the most prevalent ESBL producer in this study. *E. coli* ST10 is a widely disseminated commensal but is also linked to ESBL production and human infections (Manges & Johnson, 2012). Moreover, *E. coli* ST10 ranked among the four main STs causing *E. coli* bovine mastitis in Switzerland in 2017 (Nüesch‐Inderbinen et al., 2019). The occurrence of *E. coli* ST10 harboring *bla*_CTX‐M‐15_ among young calves highlights the potential of this lineage to disseminate into the environment via fecal shedding, with implications for bovine and human health.

Other *E. coli* included *E. coli* ST58 and ST540 harboring *bla*_CTX‐M‐1_, and ST88 carrying *bla*_CTX‐M‐3_. These three STs have previously been associated with cases of bovine mastitis (Dahmen et al., 2013; Freitag et al., 2017; Nüesch‐Inderbinen et al., 2019), and their occurrence as ESBL‐producers may have negative consequences for animal health.

Notably, all *E. coli* harboring *bla*_CTX‐M‐3_ were isolated from fecal samples of calves reared on the same farm. This suggests the horizontal spread of a mobile genetic element for example a plasmid carrying *bla*_CTX‐M‐3_ and illustrates the need to further investigate possible factors that may contribute to transmission dynamics on dairy farms.

Finally, *C. braakii* harboring *bla*_CTX‐M‐1_ was found in three calves on the same farm. This species infrequently causes infections in humans, and reports on ESBL‐producing *C. braakii* remain rare (Liu et al., 2020). CTX‐M‐producing *C. braakii* have been identified in pork in China, in fish in Tanzania (Moremi et al., 2016), and in raw milk in Germany (Odenthal et al., 2016). Thus, our data provide further evidence for the occurrence of ESBL‐producing *C. braakii* in the dairy farm environment.

This study has some limitations. The first limitation is the small number of farms and sampling occasions, and some differences between conventional and organically reared calves may have remained undetected. Second, data on AMU in the present study were communicated verbally by farm owners or staff and may have been subject to unprecise or non‐objective reporting. The number of calves with a history of AMU was very small and consequently, the results should be interpreted with caution. Finally, we did not account for additional factors that may have an impact on AMR on dairy farms, for example, by including environmental samples, and the observational nature of our study design leaves the possibility of containing confounders.

Nevertheless, the findings of this study offer new useful information with regard to developing farming management strategies that aim to mitigate the occurrence of ESBL‐producing Enterobacteriaceae on dairy farms and their dissemination to the environment and the food chain.

## 5 CONCLUSIONS

AMR was shown to be prevalent among commensal *E. coli* from young calves from both organic and from conventional dairy farms, with particular resistance phenotypes occurring more frequently in *E. coli* from organic calves. In both groups, AMR *E. coli* occurred in calves with and without AMU. The occurrence of ESBL‐producing Enterobacteriaceae was found to be significantly associated with conventionally managed farming systems. Further research on environmental and host factors that promote AMR in commensal *E. coli o*f young dairy calves is required. Factors associated with conventional dairy farming that may co‐select for the maintenance of ESBL producers should be identified.

## CONFLICTS OF INTEREST

None declared.

## ETHICS STATEMENT

This study was performed following the Swiss Animal Welfare Act (SR 455) and the involved procedures were noninvasive and are ethically accepted by the FSVO. Participation in this study was voluntary, and the farmers approved of the purpose and methods of this study. All information was treated anonymously.

## AUTHOR CONTRIBUTIONS

Magdalena Nüesch‐Inderbinen: conceptualization (supporting), methodology (supporting); visualization (lead); writing—original draft (lead); writing—review & editing (lead). Claudia Hänni: investigation (lead); methodology (lead); writing—original draft (supporting). Katrin Zurfluh: formal analysis (supporting); investigation (supporting); methodology (lead). Sonja Hartnack: formal analysis (lead); methodology (lead); supervision (supporting); writing–original draft (supporting). Roger Stephan: conceptualization (lead); investigation (lead); project administration (lead); resources (lead); supervision (lead); validation (lead); writing–review & editing (supporting).

## Data Availability

The data generated or analyzed during this study have been provided in this article.
